# Effects of ECG Data Length on Heart Rate Variability among Young Healthy Adults

**DOI:** 10.3390/s21186286

**Published:** 2021-09-19

**Authors:** En-Fan Chou, Michelle Khine, Thurmon Lockhart, Rahul Soangra

**Affiliations:** 1 Department of Biomedical Engineering, Henry Samueli School of Engineering, University of California at Irvine, Irvine, CA 92697, USA; enfanc@uci.edu (E.-F.C.); mkhine@uci.edu (M.K.); 2 School of Biological and Health Systems Engineering, Arizona State University, Tempe, AZ 85281, USA; thurmon.lockhart@asu.edu; 3 Department of Physical Therapy, Crean College of Health and Behavioral Sciences, Chapman University, Irvine, CA 92618, USA; 4 Department of Electrical and Computer Science Engineering, Fowler School of Engineering, Chapman University, Orange, CA 92866, USA

**Keywords:** autonomic nervous system, electrocardiography ECG, fluctuations, heart rate variability, nonlinear analysis, chaos

## Abstract

The relationship between the robustness of HRV derived by linear and nonlinear methods to the required minimum data lengths has yet to be well understood. The normal electrocardiography (ECG) data of 14 healthy volunteers were applied to 34 HRV measures using various data lengths, and compared with the most prolonged (2000 R peaks or 750 s) by using the Mann–Whitney U test, to determine the 0.05 level of significance. We found that SDNN, RMSSD, pNN50, normalized LF, the ratio of LF and HF, and SD1 of the Poincaré plot could be adequately computed by small data size (60–100 R peaks). In addition, parameters of RQA did not show any significant differences among 60 and 750 s. However, longer data length (1000 R peaks) is recommended to calculate most other measures. The DFA and Lyapunov exponent might require an even longer data length to show robust results. Conclusions: Our work suggests the optimal minimum data sizes for different HRV measures which can potentially improve the efficiency and save the time and effort for both patients and medical care providers.

## 1. Introduction

Heart rate variability (HRV) is a promising measure used to assess cardiovascular health by investigating heartbeat fluctuations over time. Electrocardiogram (ECG) is an autonomically controlled physiological vital signal that changes due to sympathetic or parasympathetic perturbations. As such, reduction in HRV is a well-established biomarker of diabetes [1,2], cardiovascular disease (CVD) [3,4], inflammation [5,6,7], obesity [8] and psychiatric disorders [9,10]. Over the past half-century, three groups of mathematical methods for determining HRV have been proposed (i) time domain, (ii) frequency domain, and (iii) nonlinear analyses [11].

Time- and frequency-domain HRV measures were standardized in 1996 by the task force of The European Society of Cardiology and the North American Society for Pacing and Electrophysiology [12]. Nonlinear variability measurements such as the Lyapunov exponent, fractal, entropy, and symbolic entropy are well-established [13]. However, the standardization in selecting a time series length for robustness in differentiating different populations and ailments is currently lacking. HRV measurements are influenced by data length, sampling frequency [14,15,16,17], noise [14,18] and, computation parameters used in specific methods such as the time delay and embedding dimensions [16,19,20,21,22,23]. The length of the heart-rate time series data are often considered a limitation for utilizing nonlinear analyses. The number of data points in time series is critical for nonlinear analysis since it is unknown whether fewer data sets can characterize the whole dynamics of the system.

The length of data is an essential factor considering shorter data acquisition time can improve patient throughput and efficiency of hospitals, healthcare providers, and home monitoring in general. Additionally, it improves patient’s adherence and overall experience. An essential part of the HRV analysis is knowing how many data points are needed to describe the system appropriately. An important rule of thumb suggested by researchers is to choose time series of at least 10^d^ data points with ‘d’ as the system’s embedding dimension [24]. In such a scenario, if the embedding dimension is 6, then at least one million data points are required. However, sometimes obtaining such long time series data from human subjects in controlled clinical environments is practically impossible. For example, to collect one million heartbeats, one has to record ECG continuously for about 277 h. This makes human subject data collection practically impossible. Besides, it is not known if shorter time series can accurately characterize the system’s dynamics. An optimal data length should capture the essential dynamics of the system. Thus, it is imperative to understand the relationship between the robustness of the HRV to data lengths, such that the minimum data points necessary for accurate measurements can be determined.

Traditionally in HRV analysis, the data acquisition time is set to at least 5 min [12]. A limitation to longer data acquisition is that the hydrogel layer used in ECG electrodes can degrade and lower the signal-to-noise ratio [20,25,26,27]. Some studies evaluated the influence of shorter data acquisition time in different HRV measures [28]. For instance, Munoz and coworkers suggested that some time-domain HRV measures can be reliably obtained from less than a minute of recording [29]. Others investigated the influences of data length in both time- and frequency-domain HRV measures [20,30,31,32,33]. In the time-domain analysis, the standard deviation of normal-to-normal (NN) interval (SDNN), root mean square of standard deviation (RMSSD), and the percentage of successive NN intervals greater than 50 ms in all NN internals (pNN50) have been suggested as reliable measures for 5 min data lengths. However, frequency-domain HRV measures cannot produce consistent conclusions. For nonlinear dynamics, Entropy-based HRV measures [34,35,36] have been used to differentiate patients with cardiovascular disease from healthy controls using shorter time-series data. Sample entropy (SampEn) is reported to be less dependent on data length than approximate entropy (ApEn) [35]. In conclusion, the relationship between the robustness of HRV derived by linear and nonlinear methods to the required minimum data lengths has yet to be systematically evaluated and clearly understood.

**Figure 1:**
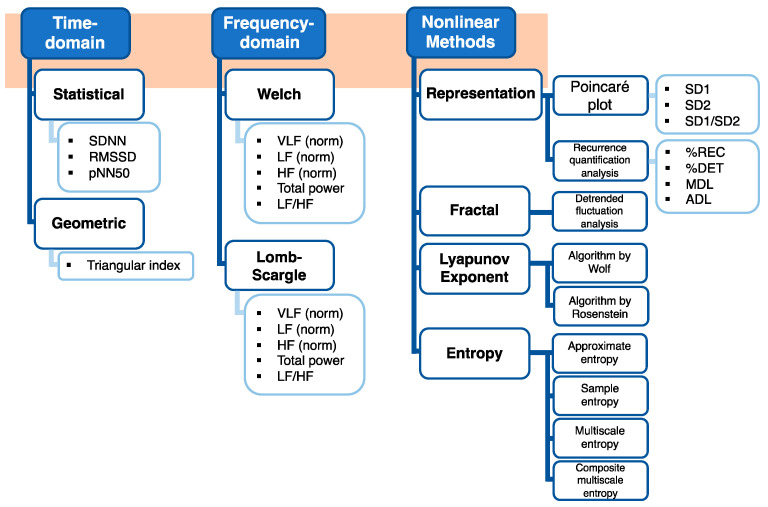
Approaches, methods, and outputted measures used to calculate HRV in the study.

In addressing such limitations, this study explores how the data length of the ECG signal affects the HRV measures (time and frequency domain variables and nonlinear variables). In this study, 14 healthy volunteers were monitored in resting-state. Various data lengths of ECG recordings were applied to eight (two time, two frequency, and four nonlinear) approaches, including 13 different methods (statistical and geometric methods in time domain, Welch’s and Lomb–Scargle in frequency domain, and Poincaré plot, recurrence quantification analysis (RQA), detrended fluctuation analysis (DFA), Wolf and Rosenstein’s Lyapunov exponents (LE), ApEn, SampEn, multiscale entropy (MSE), and composite multiscale entropy (CMSE) in nonlinear analyses), and 34 HRV measures (Figure 1), to determine the shortest data length that can keep the system dynamics intact. To understand the robustness of optimal minimum data length that can be utilized to quantify HRV, each data set size was compared with the most prolonged (2000 R peaks or 750 s) using the Mann–Whitney U test to determine the 0.05 level of significance.

## 2. Materials and Methods

### 2.1. Subjects

The data were collected from healthy young participants with normal ECG recordings. A total of 14 subjects (seven male) participated with the age of 23.8 ± 4.1 years (mean ± standard deviation) and body mass index (BMI) of 23.4 ± 5.1 kg/cm^2^. Participants were excluded if any neurological disorder or heart-related disease was reported. Only one male participant reported having a history of Kawasaki’s disease but did not present any symptoms during these data collection.

### 2.2. Experimental Protocol

The ECG signals were recorded with lead II placement at a sampling rate of 2 kHz from 14 subjects by BIOPAC MP36 System (BIOPAC Systems, Inc., Goleta, CA, USA). Subjects were asked to avoid taking caffeine two hours before the time of the study. Participants were provided enough rest on the chair (at least five minutes) after arrival to ensure the subjects were recorded in a relatively calm state during the studies. At least 10 min of ECG recordings were acquired while the subjects were seated on the chair. All subjects provided written informed consent for the study, which the Institutional Review Board of the University of California approved (IRB #2016-2924).

### 2.3. Data Preprocessing

The effect of data length on HRV measures was quantified by analyzing R-R intervals on the raw ECG recordings using time, frequency domain and nonlinear analyses methods. We segmented and standardized ECG data length using R peaks ranging from 60 to 2000, thus controlling heart rate (HR) differences [20]. In the study, three statistical (SDNN, RMSSD and pNN50) and one geometrical method (triangulation index) were included in time-domain measurements (Figure 1). Welch and Lomb–Scargle algorithms for each of the eight measures (total power, power and normalized power of very low-frequency, low-frequency, and high-frequency, and the ratio of low-frequency to high-frequency) were investigated in the frequency-domain method. Eight nonlinear methods were investigated to present different HRV measures. Besides the recurrence quantification analysis (RQA), all the measures were computed with consecutive discrete R-R intervals with R peaks ranging from 60 to 2000. RQA was estimated by raw ECG recordings ranging from 60 to 750 s. In the section below, we provided details for (i) R peak extraction, (ii) evaluation for HRV measures, and (iii) statistical analysis.

### 2.4. Extraction of R Peaks Using Wavelet Analysis

**Figure 2:**
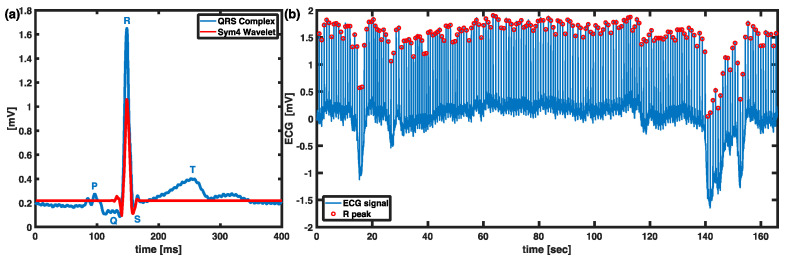
R peak detection technique. (**a**) Sym4 resembles the QRS complex that can be used for the wavelet transform. (**b**) A representative raw ECG signal with extracted R peaks in red circles.

The consecutive discrete R-R intervals were processed initially from the raw ECG signal. Symlet 4 (Sym4) wavelet was chosen to enhance R peaks by using maximal overlap discrete wavelet transform (MODWT) due to its similarity with the QRS complex as shown in Figure 2. Potential artifacts as arrhythmic events were excluded. The identified R peaks were then extracted as R-R interval segments used for the HRV analysis.

### 2.5. Time-Domain Analysis

Three statistical measures (SDNN, RMSSD, pNN50) and a geometric measure (triangular index) were discussed in time-domain analysis. SDNN is the standard deviation of the normal-to-normal (NN) interval. RMSSD represents the square root of the mean of the sum of the squares of differences between adjacent NN intervals. pNN50 calculates the ratio of the counts of adjacent NN intervals that are more than 50 ms and the total number of NN intervals in the data set. The basic variable in the geometric method, HRV triangular index, is also computed (Equation (1)).

(1) $$\mathrm{HRV}\;\mathrm{Triangular}\;\mathrm{Index}\;=\frac{\mathrm{total}\;\mathrm{number}\;\mathrm{of}\;\mathrm{NN}\;\mathrm{intervals}}{\mathrm{number}\;\mathrm{of}\;\mathrm{NN}\;\mathrm{intervals}\;\mathrm{in}\;\mathrm{the}\;\mathrm{modal}\;\mathrm{bin}}$$

SDNN, RMSSD, pNN50 and HRV triangular index were computed as standards of measurement [12].

### 2.6. Frequency-Domain Analysis

According to the task force’s [12] guideline, frequency domain measures of HRV can be categorized into four bands, ultra-low-frequency (ULF, ≤0.003 Hz), very low-frequency (VLF, 0.003–0.04 Hz), low-frequency (LF, 0.04–0.15 Hz), and high-frequency (HF, 0.15–0.4 Hz). It is also suggested that VLF, LF, and HF rhythms are distinct components for a 2–5 min short-term ECG recording. Hence, there were eight parameters calculated in the study: (i) VLF power, (ii) LF power, (iii) HF power, (iv) total power, (v) normalized VLF (VLF norm), (vi) normalized LF (LF norm), (vii) normalized HF (HF norm), and (viii) the ratio of LF to HF (LF/HF). The total power is a sum of the VLF, LF, and HF absolute power. The normalized HRV power is defined as the proportion of one power range to the total power in absolute values and allows to compare individuals and various data lengths appropriately. To estimate HRV power spectral analysis, both Welch’s method [37] and Lomb–Scargle periodogram [38,39] were applied. Welch’s method utilizes fast Fourier transform (FFT) and is advantageous for low a computation workload. The original signal with N data points is split into K segments, $X_{1}\left(j\right),\ldots ,X_{K}\left(j\right)$. Each segment has a length of L and is apart from the previous segment in the distance of D such that N can be written as Equation (2).

(2) N=(K−1)D+L.

The overlapped segments are helpful to mitigate the loss. The windowed finite Fourier transform $A_{k}\left(n\right)$ is applied to each segment (k=1, 2, …, K) with a data window W(j) where j=0, …, L−1 as Equation (3):

(3) $$A_{k}\left(n\right)=\frac{1}{L}\overset{L-1}{\underset{j=0}{\sum }}X_{k}\left(j\right)W\left(j\right)e^{-\frac{2kijn}{L}}\;\;\;\;\;\;\;\;n=0,\;\ldots ,\;\frac{L}{2},$$

where $X_{k}\left(j\right)W\left(j\right)$ is the windowed segment sequences and $i=\sqrt{-1}$. Therefore, the modified periodogram, $P_{k}\left(f_{n}\right)$, for each segment, can be written as Equation (4).

(4) $$P_{k}\left(f_{n}\right)=\frac{L^{2}{\left|A_{k}\left(n\right)\right|}^{2}}{\sum_{j=0}^{L-1}W^{2}\left(j\right)}\;\;\;\;\;\;f_{n}=\frac{n}{L}\;\;\;\;\;\;n=0,\;\ldots ,\;\frac{L}{2}.$$

And Welch’s method is estimated as the average of the periodogram values as Equation (5).

(5) $$\langle P\left(f_{n}\right)\rangle =\frac{1}{K}\overset{K}{\underset{k=1}{\sum }}P_{k}\left(f_{n}\right).$$

The Lomb–Scargle periodogram is inspired by the Fourier transform and a least-squares method known for identifying periodicity. It offers advantages in dealing with unevenly sampled data and allows ectopic or missing beats [40,41,42].

### 2.7. Nonlinear Methods

We investigated several nonlinear variability methods such as Poincaré plots, fractal dimension, Lyapunov exponent, and entropy in the study.

#### 2.7.1. Poincaré Plot

**Figure 3:**
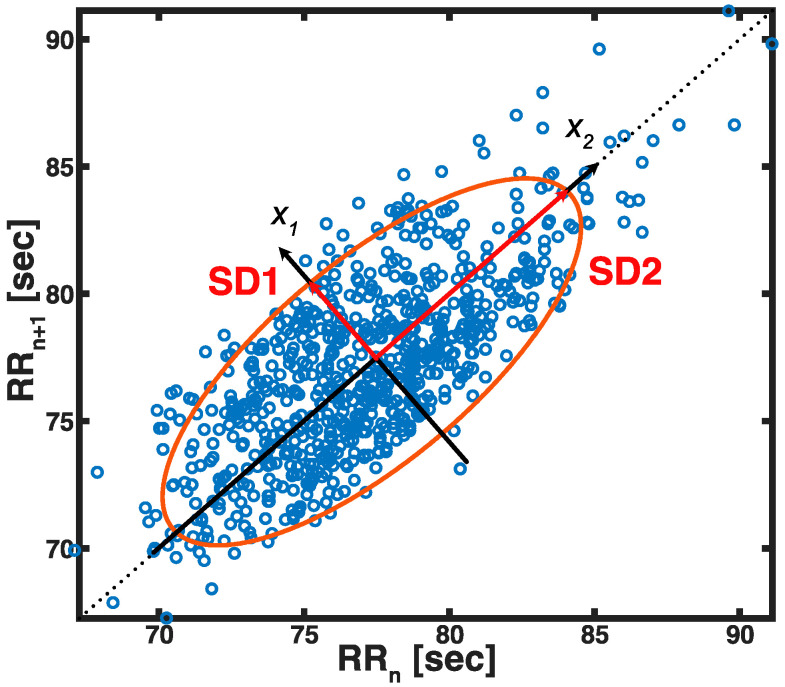
A representative Poincaré plot with a new set of a coordinate plane. The axes of the plane are $x_{1}$ and $x_{2}$. SD1 and SD2 represent the radii of a fitted ellipse on $x_{1}$- and $x_{2}$-axis.

The Poincaré plot is a nonlinear technique that can depict HRV in a two-dimensional graphic. It visualizes the beat-to-beat detail to dispersion and provides quantitative information on cardiac performances. Popular approaches to quantify Poincaré plot include ellipse fitting, histogram, and correlation coefficient. The study characterized the R-R interval as illustrated by the Poincaré plot by fitting an ellipse as in Figure 3 [43,44,45].

A new set of the coordinate plane, $x_{1}$ and $x_{2}$, is formed at the intersection of the ellipse center (Equation (6)).

(6) $$\left[\begin{array}{c} x_{1}\\ x_{2} \end{array}\right]=\left[\begin{array}{cc} \cos\theta & -\sin\theta \\ \sin\theta & \cos\theta \end{array}\right]\left[\begin{array}{c} RR_{n}\\ RR_{n+1} \end{array}\right]$$

SD1 and SD2 represent the distribution of points perpendicular and parallel to the line-of-identity of the fitted ellipse, which indicates the level of short- and long-term variability [46,47]. Mathematically, SD1 and SD2 are the standard deviations around $x_{1}$ and $x_{2}$, respectively. They were computed by using linear measures of HRV as shown in Equations (7) and (8) [47]:

(7) $$SD1^{2}=Var\left(x_{1}\right)=Var\left(\frac{1}{\sqrt{2}}RR_{n}-\frac{1}{\sqrt{2}}RR_{n+1}\right)\;=\frac{1}{2}Var\left(RR_{n}-RR_{n+1}\right)=\frac{1}{2}SDSD^{2}$$

(8) $$SD2^{2}=2SDNN^{2}-\frac{1}{2}SDSD^{2},$$

where the standard deviation of the differences between adjacent RR intervals is denoted by SDSD. The axis ratio SD1/SD2 indicates the relationship of the instantaneous interval variation to the long-term variation.

#### 2.7.2. Approximate Entropy

Approximate entropy (ApEn) is a method to quantify the complexity of time-series data. Its application is limited to data lengths greater than 100 data points [48,49]. Complexity can quantify variability to indicate the unpredictability of HR fluctuations. To assess complexity, ApEn was computed as the difference between the probability of the series of a vector with a fixed data length m and the probability of the series of another vector with a similar length (m+1) that both fall within a tolerance r as Equation (9):

(9) $$ApEn\left(m,\;r,\;N\right)=\Phi^{m+1}\left(r\right)-\Phi^{m}\left(r\right),$$

where $\Phi^{m}\left(r\right)$ from the element in Eckmann–Ruelle (E–R) entropy [50] is defined in Equation (10):

(10) $$\Phi^{m}\left(r\right)=\frac{1}{N-m+1}\overset{N-m+1}{\underset{i=1}{\sum }}logC_{i}^{m}\left(r\right),$$

where $C_{i}^{m}\left(r\right)$ is the conditional probability of vector length m.

Pincus et. al. recommended that given 1000 data points, using parameters m=2 and an r value between 0.1 and 0.25 of data standard deviation provide a robust result of ApEn [48,51]. Hence, ApEn was applied to different quantities of R-R interval time series with the values m=2 and r=0.2 in this study.

#### 2.7.3. Sample Entropy

Sample entropy (SampEn) was initially introduced 9 years after ApEn [52]. With a similar goal, SampEn is a useful mathematical algorithm that measures the predictability in time series and can be viewed as a refinement of ApEn. SampEn addressed ApEn’s shortcoming as its ability to have independence for different data lengths and showed robustness for the change in data lengths [52,53,54]. Richman and Moorman defined the negative natural logarithm of the probability using vector length m, tolerance r, and signal data length N from ApEn (Equation (11)):

(11) $$SampEn\left(m,\;r,\;N\right)=-ln\frac{A}{B},$$

where A and B are $\frac{\left(N-m\right)\left(N-m-1\right)}{2}$ times the sum of all the conditional probabilities, $C_{i}^{m+1}$ and $C_{i}^{m}$, divided by N−m without considering the self-matches.

#### 2.7.4. Multiscale Entropy

The traditional multiscale entropy (MSE) algorithm [55,56] is conducted in two parts: (i) a coarse-graining procedure for different scaled time series from an original signal; (ii) SampEn is used to calculate each time series scale. However, some disadvantages of traditional MSE had been issued [57]. Therefore, several newly developed MSE methods were reported [29,58,59,60]. However, most of the solutions suggested modifying the first step’s time-series scale with different algorithms or using other entropies for the second step instead [61]. Therefore, instead of the conventional MSE, a composite multiscale entropy (CMSE) [60] was used to evaluate cardiovascular complexity in the study. Traditional MSE computed SampEn with the first coarse-grained time series of each scale only (Equation (12)):

(12) $$SampEn\left(m,\;r,\;N\right)=-ln\frac{A}{B},$$

where x is the original one-dimensional time series, τ is the scale factor, and the same parameters m and r from computing SampEn. Equation (13) is the first coarse-grained time series $y_{1}{}^{\left(\tau \right)}$ which is defined as:

(13) $$y_{1,j}{}^{\left(\tau \right)}=\frac{1}{\tau }\overset{j\tau }{\underset{i=\left(j-1\right)\tau +1}{\sum }}x_{i},\;\;\;\;\;\;\;\;\;\;\;\;\;1\leq j\leq \frac{N}{\tau }.$$

Nevertheless, the SampEn of the first coarse-grained time series derives poor reliability. The CMSE algorithm was then introduced to overcome the problem [60]. Therefore, instead of using the first coarse-grained time series, All the coarse-grained time series SampEn are considered in CMSE as shown in Equation (14):

(14) $$CMSE\left(x,\;\tau ,\;m,\;r\right)=\frac{1}{\tau }\overset{\tau }{\underset{k=1}{\sum }}SampEn\left(y_{k}{}^{\left(\tau \right)},\;m,\;r\right),$$

where $y_{k}{}^{\left(\tau \right)}$ represents the coarse-graining procedure determined from $x_{k}$, the kth data point from the original signal (Equation (15)).

(15) $$y_{k,j}{}^{\left(\tau \right)}=\frac{1}{\tau }\overset{j\tau +k-1}{\underset{i=\left(j-1\right)\tau +k}{\sum }}x_{i},\;\;\;\;\;\;\;\;\;\;\;\;\;\;1\leq j\leq \frac{N}{\tau },\;1\leq k\leq \tau .$$

#### 2.7.5. Detrended Fluctuation Analysis

In the time series analysis, the detrended fluctuation analysis (DFA) is used to estimate self-similarity. It is calculated as the root-mean-square error of the least-squares line fitted in separate non-overlapping windows of the cumulative integral of the original time series. For example, given the interbeat intervals of an original ECG signal in time series x with length N, the cumulative integral of the signal y(k) is written as Equation (16):

(16) $$y\left(k\right)=\overset{k}{\underset{i=1}{\sum }}\left(x\left(i\right)-\langle x\rangle \right),$$

where 〈x〉 denotes the average of x, y(k) is then divided into segments of equal length n. The root-mean-square fluctuation F as a function of the window sizes n is computed by Equation (17):

(17) $$F\left(n\right)=\sqrt{\frac{1}{N}\overset{N}{\underset{k=1}{\sum }}{\left[y\left(k\right)-y_{n}\left(k\right)\right]}^{2},}$$

where $y_{n}\left(k\right)$ is the y-coordinate of the linear fitted line in each of the segments.

The fluctuation is indicated as the slope of F(n) versus n in a logarithmic scale by a scaling exponent α. Peng’s work found that there is a significant difference in α over a wide range of window sizes (20≤n≤1000) among the interbeat interval time series of 15 severe heart failures and 12 controls [62].

#### 2.7.6. Recurrence Quantification Analysis

The research of recurrences is commonly used to understand the complexity of a nonlinear dynamical system. The RQA is known for being able to handle short and nonstationary data. The recurrence plot (RP) and its quantification measures are the tools to visualize and quantify the recurrence behavior of the state space trajectory [63,64,65]. RP was first introduced in 1987, allowing the recurrences of higher dimensional phase space to be visualized by a two-dimensional representation [66]. It is an effective way to understand the behavior of a dynamic system. Mathematically, it shows the phase space vectors $\vec{x_{i}}$ that recur at time i and another time j (Equation (18)):

(18) $$R_{i,j}=\Theta \left(\varepsilon_{i}-\left|\left|\vec{x_{i}}-\vec{x_{j}}\right|\right|\right),\;\;\;\;\;\;\vec{x_{i}}\;\in R^{m},\;\;\;i,\;j=1,\ldots ,\;K,$$

where K is considered size for the recurrence matrix, m is the highest dimension being investigated, $\varepsilon_{i}$ is a threshold distance, and Θ(.) is the Heaviside function.

To infer RQA, there are three parameters that need to be taken into consideration, a time delay τ, the embedding parameter D, and a threshold distance ε. The time-delay parameter of the Takens embedding theorem [67] brings the original one-dimensional time series into multiple dimensional manifolds. Here, the average mutual information (AMI) is used to estimate τ [68]. Essentially, AMI calculates the least dependent information in the time-delayed coordinates. Then, the embedding parameter comes in to reconstruct the phase space vector since a time delay is applied to the raw data. The choice of a deficient D may lead to unwanted results such as the false bifurcation points [68]. Hence, false nearest neighbors [69], which inspects whether there’s a significant change in the distance between two adjacent data points with embedding dimensions, was used to determine D here. In our work, the time delay and embedding parameters were chosen as the median values across 14 studies (τ=22 and D=2). Lastly, the threshold distance parameter defines who the RP neighbors are as the radius of a sphere. To select a sufficient threshold, distance can be critical, it may lose the key information of the recurrence structure or include a lot of artifacts if ε is chosen too small or too large [13,70]. It is suggested that a proper selection of ε should correspond to a specific range of the percent recurrence (%REC), a quantification measure is discussed in detail in the next paragraph [64,71].

In comparing various subsets of data, the recommended threshold parameter should be chosen so that a typical %REC is in the range of 5% to 10% and the minimum is at least 1% [71]. The threshold of 8 fulfilled the guideline in the study. The quantification measures are used to characterize the information in RPs. Four of them, %REC, percent determinism (%DET), the average diagonal line length (ADL), and the maximum diagonal line length (MDL), were reported in the paper. %REC quantifies the percentage of recurrent points in a RP (Equation (19)).

(19) $$\%\mathrm{REC}\;=\frac{\mathrm{sum}\;\mathrm{of}\;\mathrm{recurrent}\;\mathrm{points}}{\mathrm{size}\;\mathrm{of}\;\mathrm{RP}}*100.$$

The minimum (0%) and maximum (100%) represent that no points and all the points are fallen into the ε-defined recurrent sphere, respectively. The second parameter, %DET, measures the percent of recurrent points occurring in connected trajectories, which is of the total counts of recurrent points (Equation (20)). The connected trajectories are formed by the continuous adjacent of two or more points that follow the diagonal lines.

(20) $$\%\mathrm{DET}\;=\frac{\mathrm{sum}\;\mathrm{of}\;\mathrm{diagonally}\;\mathrm{adjacent}\;\mathrm{recurrent}\;\mathrm{points}}{\mathrm{sum}\;\mathrm{of}\;\mathrm{recurrent}\;\mathrm{points}\;\mathrm{in}\;\mathrm{RP}}*100.$$

ADL calculates the average length of connected trajectories. MDL simply counts the length of the longest connected trajectory in the RP.

#### 2.7.7. Lyapunov Exponent

The Lyapunov exponent (LE) of a dynamical system is a quantity that measures how fast two infinitesimally close trajectories separate in phase space based on the initial condition. For instance, one point would exponentially diverge from another if the system is chaotic. Given two close trajectories, x(t) and y(t) are a function of time, Equation (21) shows the next iteration ε after time t separates their distance exponentially:

(21) $$\left|x\left(t+\varepsilon \right)-y\left(t+\varepsilon \right)\right|=\left|x\left(t\right)-y\left(t\right)\right|e^{\lambda_{1}\varepsilon },\;$$

where $\lambda_{1}$ represents as the first LE (Equation (22)). Hence, the first LE can be written as:

(22) $$\lambda_{1\;}=\underset{\varepsilon \to \infty }{lim}\underset{\left|x\left(t\right)-y\left(t\right)\right|\to 0}{lim}\frac{1}{\varepsilon }ln\left(\frac{\left|x\left(t+\varepsilon \right)-y\left(t+\varepsilon \right)\right|}{\left|x\left(t\right)-y\left(t\right)\right|}\right).$$

The rate of separation, LE, may vary for different orientations of the initial two close trajectories. λ with a positive value (λ>0) means the trajectories diverge exponentially. On the contrary, two nearby points converge exponentially, which leads to a negative value of λ (λ<0). That is to say, the larger the value of LE, the lower the predictability for a dynamical system. The largest Lyapunov exponent (LLE) is commonly referred to as the indicator of chaos. There are various computational methods to quantify LLE. Two widely used algorithms, Wolf and Rosenstein methods, are included in the study. Both of them track the divergence of nearest neighbors over time. However, Wolf’s algorithm [72] takes one trajectory as the reference only (Equation (23)). Thus, each point on the reference trajectory iterates with its single nearest neighbor’s trajectory over time until their distance apart from each other grows beyond a threshold:

(23) $$\left|\left|z\left(t_{i}\right)-x\left(t_{i}\right)\right|\right|=L\left(i\right),$$

where i is the increment, L is the difference in two trajectories, x is the point on the reference trajectory, and z is the nearest neighbor of the corresponding point. The reference point re-evaluates the new single nearest neighbor once the previous trajectory’s separation is large. Following the nearest neighbor and replacing it with another trajectory completes at the end of the reference trajectory. The distance between the reference point and the beginning and last point of each new nearest neighbor trajectory is denoted as L and $L^{\prime }$, respectively. The LLE by Wolf’s algorithm, $\lambda_{1}$, tracks all the L and $L^{\prime }$ (Equation (24)):

(24) $$\lambda_{1}\;≃\frac{1}{K}\overset{M-1}{\underset{i=0}{\sum }}ln\frac{{L^{\prime }}_{i}}{L_{i}},$$

where M represents the number of nearest neighbor trajectories and K is the total number of iterations in the reference trajectory.

Instead of focusing on a single nearest neighbor, Rosenstein’s method [73] finds the nearest neighbor overall points on the trajectory. The distance of a certain reference point, $X_{j}$, to its nearest neighbor ${X_{j}}^{\prime }$ can be expressed as Equation (25):

(25) $$d_{j}\left(0\right)=min_{{X_{j}}^{\prime }}\left|\left|X_{j}-{X_{j}}^{\prime }\right|\right|,$$

where $d_{j}\left(0\right)$ means the initial distance between the jth point and the nearest neighbor. The LLE by Rosenstein’s work can be estimated as the average speed of nearest neighbor separation (Equation (26)).

(26) $$d_{j}\left(i\right)\approx C_{j}e^{\lambda_{1}\left(i\Delta t\right)},$$

where Δt is the period of each iteration, i is the number of iterations, and $C_{i}$ as the initial separation.

In our approach, the original one-dimensional signal was reconstructed using the methods mentioned above to define parameters, time delay τ and embedding dimension D (details in RQA section). To compare different data lengths, each τ and D in a particular data length group was obtained as the median values across all participants. The selected values of τ and D in each group are referenced in Table 1.

### 2.8. Statistical Analysis

A total of 34 HRV indices were computed in this study. The indices were divided into three groups: the effect of data length on (i) time domain, (ii) frequency domain, and (iii) nonlinear HRV measures. Descriptive data are presented as means and standard deviations (SDs) for continuous HRV variables. Normal distribution of all time/frequency domain, linear/nonlinear variables was tested using the Kolmogorov–Smirnov test and visually inspected histograms and Q-Q plots. HRV parameters from ECG recordings did not exhibit normal distributions and were analyzed as non-parametric. To understand the robust/optimal minimum data length that can be utilized to quantify HRV in the methods as mentioned above, each data set size was compared against the most extended (2000 R peaks or 750 s) using the Mann–Whitney U test in R. Moreover, HRV measures were then calculated with several randomly chosen segments and compared against each other using the Mann–Whitney U test to eliminate the possibility of biased results from short data length selection. The critical value was chosen at the 0.05 level of significance.

## 3. Results

### 3.1. Time-Domain HRV

Among the four time-domain HRV measures presented in the paper, we find SDNN, RMSSD, and pNN50 are consistent for a very short-term HRV analysis (Table 2). SDNN in 100 R peaks was not significantly different from SDNN at 2000 R peaks. Similarly, a minimum of 60 R peaks of RMSSD and pNN50 were found consistent with longer (2000 R peaks) HRV recordings. The HRV triangular index, however, is recommended to be used with at least 1000 R peaks. This means that if one were an adult with a normal resting HR, a 10 to 16-min recording would have no statistically significant difference compared with a 20 to 33-min recording.

### 3.2. Frequency-Domain HRV

Both Welch and Lomb–Scargle periodograms were included in the HRV power spectral analysis (Table 2). With Welch’s method, the appropriate minimum length for acquiring VLF power, LF power, total power, and VLF norm was 1000 R peaks. The HF norm analysis can use 750 R peaks. The HF power, LF norm, and LF/HF ratio had no change from 60 to 2000 R peaks. On the contrary, the lengths required to calculate HRV measures by the Lomb–Scargo periodogram were shorter than Welch’s in general. However, the recommended lengths to obtain VLF norm, LF norm, and LF/HF ratio remained same. Furthermore, using 200, 500, 500, and 750 R peaks for HF norm, LF power, total power, and HF power, respectively, were acceptable. VLF power with the Lomb–Scargle algorithm had no statistically significant difference using very short-term recordings.

### 3.3. Nonlinear HRV

The statistical results of nonlinear HRV indices were separated into two tables. The majority of nonlinear HRV indices are shown in Table 2. The measures of RQA are listed in Table 3. As a whole, the adequate lengths of nonlinearly-assessed HRV measures were longer than those of linearly-assessed methods.

Poincaré plots, when compared with different data lengths, did not show any differences with SD1. However, the minimum lengths of 1000 and 750 R peaks were not significantly different compared with the maximum (2000 R peaks) for SD2 and SD1/SD2, respectively (Table 2). Furthermore, as entropy-based approaches, using 750 R peaks to quantify SampEn, ApEn, and CMSE showed no significant differences compared with maximum length (2000 R peaks); however, traditional MSE was significantly different until 750 R peaks and was robust for longer data lengths. Statistical results showed that DFA and Wolf and Rosenstein’s LE were affected due to the data length. The minimum data length for DFA and Wolf’s LE was 1500 R peaks such that no significant difference was found compared with 2000 R peaks (maximum data length). However, Rosenstein’s LE showed significantly different values in all the data lengths when compared with full data length (2000 R peaks). Four parameters of RQA (Recurrence (REC), determinism (DET), Maximum Diagonal Length (MDL) and Average Diagonal Length (ADL)) were computed for different data lengths to compare with the maximum (ECG data of 750 s or 12.5 min) data. The results did not show any significant differences among 1 min and 12.5 min of %REC, %DET, MDL and ADL.

Table 4 shows recommended minimum data length suggesting consistency and unbiased to maximum data length (2000 R peaks) using the Mann–Whitney U test. We reported time- and frequency-domain HRV measures and minimum data length with consistency. We found that most of nonlinear variables required a minimum data length of 1000 or 1500 R peaks. However, this was not the case for SD1 of Poincaré plot and RQA measures.

**Figure 4:**
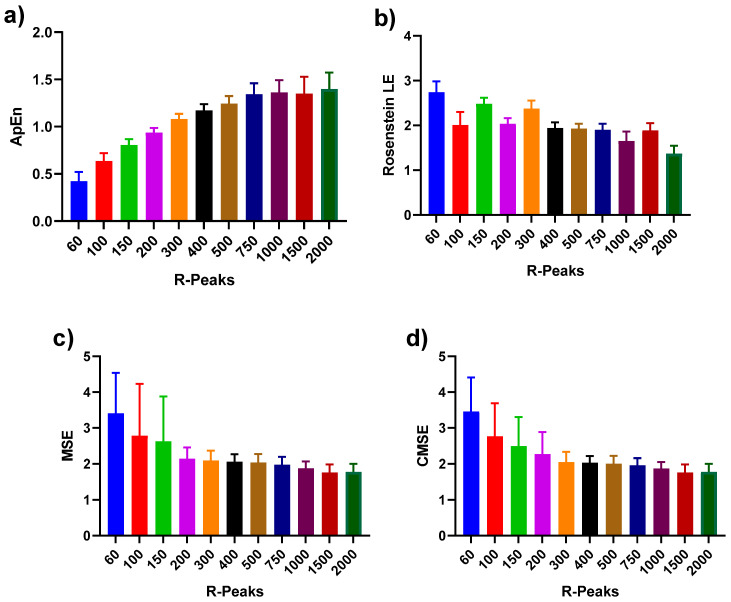
Bar plots of (**a**) ApEn, (**b**) Rosenstein’s LE, (**c**) MSE, and (**d**) CMSE with different numbers of R peaks. Different R-peaks are represented with different colors for four nonlinear methods. The error bars represent standard deviation (SD) of the values among 14 participants.

We investigated the effects of data length HRV measures on 14 participants utilizing the Mann–Whitney U test. Box plots were used to summarize the distribution in each HRV measure per data length (Figure 4). Figure 4a shows how data length affects the complexity (ApEn) of the R-R interval data. With the data length increasing from 60 to 2000 R peaks, the overall value of ApEn showed a linear trend and reached a plateau around 750 R peaks. The median values and the 25–75 percentile range among 750 to 2000 R peaks remained consistent, contrasting with 60 to 500 R peaks (showed increasing linear trend). Unlike the pattern in ApEn, Rosenstein’s LE showed inconsistency while the data lengths increased. In Figure 4b, the box plots of Rosenstein’s LE show scattered values with the observation of the outlier quantity. Both MSE (Figure 4c) and CMSE (Figure 4d) had more extensive percentile ranges in shorter data lengths and a similar variation in median values after 200 R peaks.

## 4. Discussion

In this study, we investigated the effects of ECG data series length on the consistency of HRV parameters. Conventionally, short assessments of five minutes of ECG data are used for analysis (approximately 360 R-R intervals) [12], and in this study, we evaluated the statistical differences of HRV measures at different data lengths to maximum data length (2000 R peaks). We investigated 34 HRV measures in this study, including (a) time domain, (b) frequency domain, and (c) nonlinear analysis variables. We found that a length of 1000 R peaks or more can precisely estimate HRV for time and frequency domain variability features. Moreover, we found all variables were affected by ECG data length. For example, in the general frequency domain variables are more unstable for up to 750 R peaks of data length.

### 4.1. Use of HRV Measures in Pathology Differentiation

Previous studies suggested that HRV measures can be an indicator to differentiate the pathologies from the healthy controls or to predict the severity of disease. Patients with common neurodegenerative diseases such as Alzheimer’s disease and Parkinson’s disease were found to have significantly reduced time- and frequency-domain HRV measures regardless of the data length [74,75,76,77]. Valappil and coworkers discussed patients with REM sleep behavior disorder; considering premotor Parkinson’s disease, they used five-minute ECG recording and found significantly lower HRV in SDNN, pNN50, LF, HF, SD1 and SD2 in the Poincaré plot compared with controls [78]. The interest in studying the use of HRV measures in other pathologies is also widespread. For instance, linear and nonlinear variability such as complexity, measured by SampEn, reduced in patients with type 1 diabetes mellitus using 3200 R peaks [79]. Mussalo and coworkers demonstrated that both patients with mild and severe essential hypertension had lower time- and frequency- domain HRV measures computed with at least 250 R peaks in the 10-min recordings [80]. In addition, there was the study presented that a five-minute ECG recording can show differences in nonalcoholic fatty liver disease with diabetes and controls [81]. Most of the studies had focused on whether linear HRV measures can stratify pathologies. To further understand how to assess not only linear but nonlinear HRV in a short time, it is crucial to investigate the effect of data lengths.

### 4.2. Importance of Short Data Sets and R-R Intervals

Various studies have demonstrated very short-term (less than 5 min) or short-term (5 min) data lengths for HRV analysis [20,29,30,31,32,34,75,77,78,79,82,83,84,85]; others have vouched for using longer data lengths for HRV analysis [74,76,86]. However, the knowledge of how data length affects different HRV linear and nonlinear measures is presently unknown. This research is essential since variation in ECG recording length may result in differences in outcomes of HRV analysis in all temporal, frequency-based and linear/nonlinear analyses. When recordings with different duration are compared, it should be considered to use the most prolonged duration as a standard of comparison for stable HRV values. This allows us to identify the minimum length of ECG data that can capture system dynamics without significantly differing results from long data sets. A quick HRV analysis may serve as a promising diagnostic tool in healthcare. An effort to shorten ECG data recording is critical since HRV features add essential information for cardiac functioning. Our study highlights that most of the HRV measures are sensitive to changes in the data length. We found the sensitivity of each HRV measure was affected by the change in data lengths. It is important to note that a faster HR leads to a smaller HRV [20]. Hence, unlike most others using time as the length reference, the quantity of R peaks ranging from 60 to 2000 was used instead to avoid HR variation [87].

### 4.3. Linear ECG Variability Measures

Chen and coworkers conducted a study with 3387 adult participants with ECG recordings of longer than two minutes, but reported that such long ECG recording may not be required since the valid results of RMSSD and SDNN can be attained from 10 and 30 s of ECG recordings, respectively [29]. The robustness of RMSSD from 10 s recordings was also corroborated in Thong’s work [30]. However, 10 s based SDNN assessment was found inconsistent by both studies concluding that linear variability measure such as SDNN was more sensitive to data length than RMSSD. Similarly, some other studies reported similar results of RMSSD and SDNN when comparing data lengths of 3 and 5 min [20], 50-s [31] and 5-min [32,83,84] measurements as the reference. On the other hand, pNN50 evaluated from three and five minutes showed similar values [20]. Short ECG data sets of 20 s of pNN50 can reliably estimate similar to 150 s [31]. Thus, our results indicate that RMSSD and pNN50 were the least sensitive to data lengths. A shorter ECG data length of 30 s for SDNN evaluation can potentially replace existing guidelines by the task force [12]. The HRV triangular index was affected by data length, which was consistent with previous findings [83].

### 4.4. Frequency-Domain Analysis

The frequency-domain HRV measures were evaluated as per standard techniques defined by the task force [12]. We investigated two frequency domain HRV analysis methods using the Welch periodogram and Lomb–Scargle. Previously, Thong investigated 10-s HRV data for HF band variables and reported results unreliable for accuracy [30]. McNames and Aboy concluded that the performances of HF variable ranging from 10 s to 10 min compared with the 5-min estimation were comparable with the results in mean HR [83]. In addition, the study showed that 40-s HF and 50-s LF/HF, LF norm and HF norm were reliable to monitor mental stress under a mobile setting [31]. Similar to Salahuddin’s work, we found 60 R peaks (36- to 60-s) LF/HF and LF norm had no significant differences with 2000 R peaks using either Welch or Lomb–Scargle algorithm. In addition, the task force manual suggested that it can be inappropriate to assess VLF in short-term recordings (≤5 min). However, our findings show that the optimum data length to estimate VLF depends on the methods of power spectral density (for example, 1000 and 60 R peaks in using the Welch and Lomb–Scargle algorithm, respectively).

### 4.5. Nonlinear Variability Analysis

Most of the nonlinear methods were proposed 30 years ago. However, there are not literatures investigating the sensitivity on data length as the linear HRV analysis. A shorter data length of Poincaré plot, MSE, and CMSE were reported and discussed for different purposes without providing any suggestions to minimum data length for reliable measurements. For instance, the short-term assessment of the Poincaré plot was applied in different stress levels, yet was concluded as promising results [88,89]. For an entropy-based HRV analysis, SampEn and ApEn were compared and discussed together in most instances since SampEn was introduced to improve the unreliable outcome of ApEn due to data length. It was suggested that a minimum data length of 100 and 250 RR intervals of SampEn and ApEn can distinguish healthy from congestive heart failure patients [34]. Another group studied in the range of 2 min to the 20 min data length. The authors concluded that SampEn was a lot less sensitive to data length compared with ApEn [35]. McNames and Aboy considered several time domain and frequency domain HRV variables with ApEn and indicated that ApEn was the most unreliable one [83]. Although, SampEn was reported independent of data length compared with ApEn. However, we found 1000 R peaks to be optimum for estimating ApEn or SampEn.

The results of DFA (α) represent the relationship of F(n) and the window sizes n. Obviously, n cannot be larger than the length of the data set. Therefore, the data length is an essential factor of the accuracy of α. Moreover, a crossover phenomenon was observed when DFA was proposed [62]. Therefore, Peng suggested that at least a 24-h recording was required for diagnostic purposes since the crossover phenomena can play an essential role consistent with our results.

To calculate LLE, the parameters τ and D need to be defined first. In this study, both τ and D increases with the data lengths in HRV. Gao’s works suggested that D=2 should be used when analyzing a finite HRV data set [90,91,92]. However, others reported larger parameters for longer data sets and shorter parameters for shorter data sets. For instance, Signorini and Cerutti calculated long-term HRV (N=20,000) with τ=7 and D=10 [86]. Li’s group used τ=1 and D=3 for less than 5 min data sets (N=200−355) [85]. Moreover, regarding the method differences in LE, Rosenstein’s LE is fast and easy to implement and applicable to small data sets [73]. A minimum of 200 consecutive R-R intervals was suggested as the optimum data length for calculating HRV [85]. However, Li and coworkers did not report any statistical evidence supporting their conclusion. On the contrary, we found that data length is significantly different from LLE of 2000 R peaks.

The varying data lengths required for nonlinear and chaos HR analyses can be partially explained by different autonomic heart control system aspects that complexity (ApEn, SampEn and MSE) and LLE can measure. For instance, MSE can measure deviations or differences at different time scales thus offering insightful information on temporal dynamical variations in the autonomic HR control system. In our laboratory pilot studies we found strenuous exercises significantly decreased the complexity of HR R-R interval data. However, LLE detects the presence of chaos in heart dynamical control systems by quantifying LEs (exponential divergence of initially close state space trajectories). The computation of LLE utilizing multiple LEs derived from divergence curves requires larger data sets for stable values. Additionally, DFA is an indicator of statistical persistence and antipersistence of HR time series. Persistence indicates the deviation in HR time series is statistically more likely followed by subsequent deviation in the same direction (increase in HR is followed by a subsequent increase in HR or decrease is followed by another decline of HR). On the other hand, antipersistence implies that the deviation is followed by subsequent deviation in the opposite direction (increase in HR is followed by a decrease in HR and vice-versa). Since supraspinal mechanisms involuntarily control HR, it is likely the HR control mechanism will naturally produce long-range correlated HR time series, thus requiring at least 1500 data points for robust and stable DFA values.

### 4.6. Limitations

The findings of this study must be seen together with the limitations. Firstly, our study is limited with sample size. Additionally, the subjects were within a limited range of age thus the study is limited in external validity to other age ranges. We will conduct a study with a larger number of participants and broader age ranges in the future. Secondly, this study investigated optimum data length for HRV measures only limited to a healthy group. Hence, a future study with pathological groups would be interesting to embolden our findings.

## 5. Conclusions

An ECG-based analysis of cardiac rhythm is critical for the diagnosis of a heart condition and disease management. In addition, novel clinical decision support systems require quick ECG analysis to assist clinicians. Our effort to shorten the ECG recording duration is vital to improve efficiency and save time and effort for patients and clinical care providers. This can be more critical for patients with frequent artifacts and HRV physiological features extracted from short ECG recordings with high confidence. Our study suggests that ECG data length collected from wearable devices must be optimized and selected such that more consistent and reliable results can be attained with existing laboratory-grade measurements. In conclusion, this study suggests ultra-short data sequences can be collected and analyzed for quick HRV assessments retaining the rich information from linear/ non-linear variability structure, but with caution since HRV variables are affected differentially to the data length. Chaotic HR analyses such as LLE (Rosenstein and Wolf) and long-range correlation through DFA required longer data set lengths than other nonlinear variability measures such as ApEn, SampEn, and MSE.

## Figures and Tables

**Table 1:** The values of time delay and embedding dimension used in different data lengths.

Length (R Peaks)	Time Delay	Embedding Dimension
60	2	2
100	3	2
150	2	3
200	3	3
300	2	3
400	3	4
500	3	4
750	3	4
1000	4	4
1500	3	4
2000	5	5

**Table 2:** Mann–Whitney U test results for comparing HRV measures at 2000 R peaks with shorter data lengths. Data length is in R peaks. Statistical significant differences (*p* < 0.05) and statistical highly significant differences (*p* < 0.001) are color labeled in lighter and darker gray with bold font, respectively.

Length	60	100	150	200	300	400	500	750	1000	1500
Time-domain HRV										
Geometric measure										
Triangular index	**0.000**	**0.002**	**0.004**	**0.004**	**0.005**	**0.008**	**0.017**	**0.048**	0.148	0.800
Statistical measure										
SDNN	**0.016**	0.056	0.056	0.069	0.056	0.062	0.104	0.094	0.265	0.946
RMSSD	0.982	0.804	0.667	0.734	0.734	0.701	0.701	0.635	0.769	1.000
pNN50	0.946	0.909	0.730	0.872	0.836	0.765	0.909	0.836	0.909	0.982
Frequency-domain HRV										
Welch’s periodogram										
VLF	**0.000**	**0.002**	**0.002**	**0.002**	**0.001**	**0.003**	**0.006**	**0.009**	0.104	0.734
LF	**0.035**	**0.050**	0.104	0.056	**0.044**	**0.048**	**0.044**	**0.044**	0.210	0.839
HF	0.603	0.804	0.910	0.839	0.874	0.910	0.874	0.946	0.982	0.982
Total power	**0.016**	**0.039**	**0.050**	**0.050**	**0.044**	**0.035**	0.057	**0.050**	0.210	0.910
VLF norm	**0.000**	**0.000**	**0.000**	**0.000**	**0.000**	**0.001**	**0.005**	**0.009**	0.103	0.646
LF norm	0.946	0.701	0.734	0.927	0.804	0.769	0.748	0.946	0.734	0.890
HF norm	**0.008**	**0.003**	**0.008**	**0.014**	**0.019**	**0.024**	**0.044**	0.085	0.137	0.734
LF/HF	0.137	0.062	0.085	0.062	0.069	0.085	0.113	0.183	0.306	0.839
Lomb–Scargle’s periodogram										
VLF	0.945	0.121	0.188	0.105	0.256	0.306	0.418	1.000	0.069	0.728
LF	**0.000**	**0.000**	**0.006**	**0.004**	**0.013**	**0.050**	0.188	0.188	0.798	0.694
HF	**0.000**	**0.000**	**0.001**	**0.001**	**0.003**	**0.011**	**0.030**	0.112	0.982	0.963
Total power	**0.000**	**0.000**	**0.001**	**0.000**	**0.001**	**0.013**	0.073	0.140	0.645	0.890
VLF norm	**0.000**	**0.000**	**0.000**	**0.002**	**0.002**	**0.010**	0.056	**0.040**	0.358	0.804
LF norm	0.137	0.435	0.839	0.807	1.000	0.982	0.910	0.769	0.890	0.982
HF norm	**0.027**	0.077	**0.048**	0.062	0.081	0.094	0.178	0.198	0.511	0.818
LF/HF	0.839	0.541	0.482	0.511	0.520	0.401	0.520	0.804	0.734	0.982
Nonlinear HRV										
Poincaré plot										
SD1	0.667	1.000	0.890	0.963	0.908	0.874	0.910	0.769	0.854	0.910
SD2	**0.009**	**0.031**	**0.021**	**0.027**	**0.014**	**0.014**	**0.031**	**0.044**	0.198	0.910
SD1/SD2	**0.004**	**0.002**	**0.008**	**0.011**	**0.009**	**0.014**	**0.039**	0.085	0.150	0.667
Entropy										
SampEn	**0.046**	**0.009**	**0.006**	**0.007**	**0.035**	**0.021**	**0.031**	0.077	0.329	0.734
ApEn	**0.000**	**0.000**	**0.000**	**0.000**	**0.000**	**0.000**	**0.011**	0.541	0.701	0.511
MSE	**0.005**	0.125	**0.012**	**0.003**	**0.002**	**0.003**	**0.007**	**0.035**	0.164	0.839
CMSE	**0.000**	**0.002**	**0.007**	**0.002**	**0.009**	**0.004**	**0.009**	0.062	0.164	0.839
Fractal										
DFA	**0.000**	**0.000**	**0.000**	**0.000**	**0.000**	**0.000**	**0.002**	**0.000**	**0.002**	0.667
Lyapunov exponent										
Wolf	**0.000**	**0.000**	**0.000**	**0.035**	**0.000**	**0.001**	0.069	0.085	**0.002**	0.701
Rosenstein	**0.000**	**0.000**	**0.000**	**0.000**	**0.000**	**0.000**	**0.000**	**0.000**	**0.002**	**0.000**

**Table 3:** Mann–Whitney U test results for comparing measures of RQA at 750 *s* with shorter data lengths. No significant differences show in any length considered.

Length (s)	60	100	150	200	300	400	500	600
%REC	0.910	0.910	1.000	0.946	1.000	0.874	0.946	0.982
%DET	1.000	0.982	0.946	1.000	1.000	0.946	1.000	0.946
MDL	0.982	0.769	0.839	0.982	0.804	1.000	1.000	0.982
ADL	0.701	0.910	0.982	0.982	0.946	0.946	0.982	0.946

**Table 4:** The recommended minimum data length of each HRV measure.

HRV Parameters	Recommended Minimum Data Length (R Peaks)
Time-domain HRV	
Geometric measure	
Triangular index	1000
Statistical measure	
SDNN	100
RMSSD	60
pNN50	60
Frequency-domain HRV	
Welch’s periodogram	
VLF	1000
LF	1000
HF	60
Total power	1000
VLF norm	1000
LF norm	60
HF norm	750
LF/HF	60
Lomb–Scargle’s periodogram	
VLF	60
LF	500
HF	1000
Total power	500
VLF norm	1000
LF norm	60
HF norm	500
LF/HF	60
Nonlinear HRV	
Poincaré plot	
SD1	60
SD2	1000
SD1/SD2	1000
Entropy	
SampEn	1000
ApEn	1000
MSE	1000
CMSE	1000
Fractal	
DFA	1500
Lyapunov exponent	
Wolf	1500
Rosenstein	-

## Data Availability

The raw data supporting the conclusions of this article will be made available by the corresponding author upon reasonable request.
